# Algal Bloom–Associated Disease Outbreaks Among Users of Freshwater Lakes — United States, 2009–2010

**Published:** 2014-01-10

**Authors:** Elizabeth D. Hilborn, Virginia A. Roberts, Lorraine Backer, Erin DeConno, Jessica S. Egan, James B. Hyde, David C. Nicholas, Eric J. Wiegert, Laurie M. Billing, Mary DiOrio, Marika C. Mohr, F. Joan Hardy, Timothy J. Wade, Jonathan S. Yoder, Michele C. Hlavsa

**Affiliations:** 1 Environmental Public Health Div, Office of Research and Development, US Environmental Protection Agency; 2 Div of Foodborne, Waterborne, and Environmental Diseases, National Center for Emerging and Zoonotic Infectious Diseases, CDC; 3 Div of Environmental Hazards and Health Effects, National Center for Environmental Health, CDC; 4 New York State Dept of Health; 5 Ohio Dept of Health; 6 Washington State Dept of Health

Harmful algal blooms (HABs) are excessive accumulations of microscopic photosynthesizing aquatic organisms (phytoplankton) that produce biotoxins or otherwise adversely affect humans, animals, and ecosystems. HABs occur sporadically and often produce a visible algal scum on the water. This report summarizes human health data and water sampling results voluntarily reported to CDC’s Waterborne Disease and Outbreak Surveillance System (WBDOSS) via the National Outbreak Reporting System (NORS) and the Harmful Algal Bloom-Related Illness Surveillance System (HABISS)* for the years 2009–2010. For 2009–2010, 11 waterborne disease outbreaks associated with algal blooms were reported; these HABs all occurred in freshwater lakes. The outbreaks occurred in three states and affected at least 61 persons. Health effects included dermatologic, gastrointestinal, respiratory, and neurologic signs and symptoms. These 11 HAB-associated outbreaks represented 46% of the 24 outbreaks associated with untreated recreational water reported for 2009–2010, and 79% of the 14 freshwater HAB–associated outbreaks that have been reported to CDC since 1978. Clinicians should be aware of the potential for HAB-associated illness among patients with a history of exposure to freshwater.

Eleven freshwater HAB–associated outbreaks that occurred in 2009 or 2010 were reported to CDC by New York, Ohio, and Washington. Freshwater HAB-associated outbreaks were defined as outbreaks where algal blooms were noted by state health or environmental investigators.† These outbreaks resulted in at least 61 illnesses, two (3%) hospitalizations, and no known deaths. Of 58 persons who reported seeking health care, seven (12%) reported a visit to an emergency department and 34 (59%) reported a visit to another type of health-care provider. Where demographic data were available, 34 ill persons were female (59%) and 38 (66%) were aged ≤19 years (Table 1). The median duration of an outbreak (time in days from the date of first reported exposure to the date of last reported exposure) was 9 days (range: 0–44 days).§ Onset time (time in days from reported exposure to reported onset of health effects) was not available for each reported illness. Among six outbreaks, the earliest onset of signs and symptoms reportedly occurred within 1 day of exposure. Among five outbreaks, median onset was calculated based on data for 27 persons; median onset times ranged from half a day to 2 days. All 11 outbreaks were associated with recreational activities at freshwater lakes during June, July, or August.

Health effects varied among outbreaks (Table 2). Effects included dermatologic signs or symptoms such as rash, irritation, swelling, or sores (eight outbreaks); gastrointestinal signs or symptoms (eight); respiratory signs or symptoms (six); fever (five); headache (four); neurologic signs or symptoms (four); ear symptoms (five); and eye irritation (three).¶ Three routes of exposure to recreational water were reported: contact (nine outbreaks), ingestion (six), and inhalation of aerosols (four) (Table 2). In seven (78%) of the outbreaks for which contact exposure was reported, affected persons developed rash or skin irritation; in each of the outbreaks for which ingestion exposure was reported, affected persons had gastrointestinal signs or symptoms; and in three (75%) of the outbreaks for which inhalation exposure was reported, affected persons had respiratory signs or symptoms.

Cyanobacteria are common components of freshwater HABs and can produce cyanotoxins, which include potent hepatotoxins, neurotoxins, and dermatotoxins that can harm humans and animals (1). Water testing practices varied among outbreak investigations (Table 3). Eight outbreak investigations included evaluation of cyanotoxins. Detections included microcystin (eight of eight investigations), anatoxin-a (three of four), saxitoxin (two of five), and cylindrospermopsin (two of five). Reported cyanotoxin concentrations varied over time. Four outbreak investigations included detection of multiple cyanotoxins; two of these investigations also revealed a potential association with bird, fish, or dog illness or deaths. Three outbreak investigations identified cyanobacteria and two identified potentially toxic cyanobacteria without quantification. One outbreak investigation included testing for bacterial indicators of fecal contamination. *Escherichia coli* was identified and quantified; samples exceeded U.S. Environmental Protection Agency (EPA) recreational water quality criteria of 126 CFU/100 mL.**

What is already known on this topic?

Cyanobacteria can form harmful algal blooms that might produce potent toxins in surface waters. Several studies have reported adverse human health effects associated with recreational water exposure to cyanotoxins and cyanobacteria blooms.

What is added by this report?

During 2009–2010 in the United States, 11 outbreaks associated with fresh water and harmful algal blooms affected at least 61 persons, resulting in two hospitalizations and no known deaths. Among 58 persons for whom data are available, seven (12%) visited an emergency department and 34 (59%) visited a health-care provider; 66% of affected persons overall were aged ≤19 years. This report suggests that the time to onset of effects might be rapid, that children might be at higher risk for illness, and that harmful algal bloom–associated outbreaks occur during the warmer months.

What are the implications for public health practice?

Untreated recreational waters with harmful algal blooms present a potentially severe health risk to humans and animals. Environmental control and prevention of blooms is needed. Efforts to identify and correctly ascertain algal bloom associated-illness will improve case detection and contribute to the development of evidence-based prevention strategies.

## Editorial Note

Outbreaks associated with freshwater HABs previously were reported infrequently; only three were reported to WBDOSS for 1978–2008.†† Freshwater cyanobacteria blooms are most likely to form on warm, stable bodies of water that are rich in nutrients (1). All outbreaks reported for 2009–2010 occurred in northern states during June–August. The World Health Organization (WHO) has proposed cell density guidelines for cyanobacteria bloom risk levels for recreational waters: 20,000 cells/mL is associated with risk for short-term adverse health outcomes; at 100,000 cells/mL, additional risk for long-term illness exists; and cyanobacterial scum formation in bathing areas is associated with the additional risk for “potentially severe health outcomes” (2).

This report highlights the challenges of recognizing HAB-associated illness. During recreation in or alongside water with HABs, persons might experience multiple routes of exposure and multiple health effects. Reported exposure routes might reflect reported health effects rather than the true exposure route, given the difficulty of determining if exposure occurred via ingestion, inhalation, or contact. Previous reports of health effects associated with recreational water exposure to HABs include gastrointestinal, respiratory, eye, ear, and dermatologic effects; fever and neurologic effects are reported less frequently (3). The nonspecific nature of these effects might make it difficult for health-care providers to identify HAB-associated illness. Health-care providers should be aware that HAB-associated illness might present differently from other recreational water–associated illnesses and onset might occur soon after exposure. For example, four of the HAB-associated outbreaks were notable for associated neurologic symptoms or confusion/visual disturbance and HAB-associated illness might occur soon after exposure (Table 2). Among the 11 reported outbreaks, most persons reporting illness were aged ≤19 years. Children might be at higher risk for HAB-associated health effects because of more frequent exposure to and greater ingestion of recreational water (4).§§

Animal deaths associated with HABs are sentinel events signaling potential risk for human illness associated with exposure to recreational waters. In two outbreaks, dead fish were reported by beach attendees and there were possibly associated dog deaths at each site. Biologic samples from affected persons and animals can be analyzed for cyanotoxins to improve exposure assessment; however, these assays are performed only by research or specialty laboratories at this time (5–7).

It is important to maintain surveillance for HAB-associated illnesses to understand their public health impact. In the United States, cyanobacteria and their toxins are unregulated drinking water contaminants (8). No federal regulation of cyanobacteria nor cyanotoxins in drinking water or any recommended guideline values for recreational waters, no standardized methods for cyanotoxin detection in water or in biologic specimens, and no national monitoring programs for occurrence currently exist. However, many states rely on guidelines published by WHO (2); others have derived their own risk assessments and developed guidelines to support public health decision-making, such as posting advisories or closing bodies of water to any use (3,9). A summary of guidelines from different countries for exposure to cyanobacteria and their toxins was recently published (10).These guidelines and reports include action levels that might be applied by local, regional, state, or tribal entities to reduce exposure of humans and animals to cyanobacteria and their toxins. Although HABISS has been discontinued, NORS will continue to provide a mechanism for national reporting of HAB-associated outbreaks.

Testing water samples for cyanotoxins can contribute to the investigation of HAB-associated illness. Microcystins were detected during all eight outbreak investigations in which cyanotoxin testing was performed. Microcystin concentrations of ≥20 *μ*g/mL exceeded the WHO guideline for moderate health risks in four outbreaks (Table 3) (2). During investigations of these outbreaks, saxitoxin, cylindrospermopsin, and anatoxin-a also were detected. To date, there are few reports of documented human exposure to these cyanotoxins in recreational waters and there are no United States or international public health guidelines on their concentrations. Notably, neurologic effects were reported in 75% of outbreaks that included detection of known neurotoxins (anatoxin-a and saxitoxin).

The findings in this report are subject to at least three limitations. First, outbreak detection varies among localities. Second, reporting is voluntary. Finally, the reports described here likely represent underreporting of freshwater HAB-associated outbreaks.

This report represents a first attempt to summarize a group of freshwater HAB-associated recreational waterborne disease outbreaks. More resources are needed for improvements in risk characterization of cyanobacteria and cyanotoxins exposure, water monitoring for potentially toxic cyanobacteria, cyanotoxin analysis of water samples and biologic specimens, and case-finding for human illnesses associated with exposure to HABs in recreational waters. HAB-associated outbreaks will likely increase as warm eutrophic bodies of water become more common over time as predicted by development and climate projections. Better characterization of the occurrence of blooms, bloom-associated environmental conditions, and of human illness associated with exposure to algal blooms is needed to develop evidence-based prevention strategies (e.g., optimized control of nitrogen and phosphorus nutrient pollution). EPA, which regulates recreational water quality, and CDC can support local and state health jurisdictions to optimize national outbreak surveillance, and thus better inform prevention and control efforts by providing guidance on what epidemiological, clinical, and environmental data are needed to support detection and investigation.

## Figures and Tables

**TABLE 1:** Number and percentage of persons (n = 58*) affected by harmful algal bloom–associated waterborne disease outbreaks, by sex and age group — United States, 2009–2010

Characteristic	No.	(%)
**Sex**		
Female	34	(59)
Male	24	(41)
**Age group (yrs)**		
<1	0	—
1–4	4	(7)
5–9	16	(28)
10–19	18	(31)
20–49	12	(21)
50–74	8	(14)
≥75	0	—

* Three persons of unknown sex and age are not included.

**TABLE 2:** Reported exposure, health effects, and health-care use resulting from harmful algal bloom–associated waterborne disease outbreaks — United States, 2009–2010

Outbreak (by state)	Cases	Health-care use*			Reported exposure†	Reported health effects (no.)§					
	
		Health-care provider	Emergency department	Hospitalized		Gastrointestinal	General	Dermatologic	Eye/Ear	Neurologic	Respiratory
New York (Outbreak 1)	2	1	0	0	Contact			Rash (2), swelling (1), sores (1)			
New York (Outbreak 2)	2¶	0	0	0	Contact				Watery eyes (2)		Nasal congestion (2)
New York (Outbreak 3)	2	0	0	0	Contact			Rash (2)			
Ohio (Outbreak 4)	3	1	0	0	Contact, ingestion, inhalation	Abdominal cramps (1), diarrhea (1), anorexia (1)	Dizziness (1), headache (1), muscle aches (1), fatigue (1), sore throat (2)	Rash (1), skin irritation (1)		Neurologic symptoms (1)	Cough (1), congestion (1), wheezing (1), shortness of breath (1)
Ohio (Outbreak 5)	19	19	0	0	Contact, ingestion, inhalation	Vomiting (11), nausea (11), abdominal cramps (7), diarrhea (5)	Fever (2)	Rash (6)	Eye irritation (5), earache (5)		
Ohio** (Outbreak 6)	7††	2	0	0	Contact, ingestion, inhalation	Anorexia (2), diarrhea (1), nausea (1)	Fever (2), fatigue (2), headache (1), muscle/joint pain (1), malaise (1), weakness (1), sore throat (1)	Rash (6), skin irritation (1)	Visual disturbance (1), earache (1)	Confusion (1)	Cough (1), wheezing (1)
Ohio (Outbreak 7)	9	3	2	0	Contact	Abdominal cramps (3), diarrhea (3), nausea (3), vomiting (2)	Fever (2), headache (2)	Rash (8)	Eye irritation (1), earache (1)	Neurologic symptoms (2), tingling (2), confusion (1)	Respiratory symptoms (1)
Ohio§§ (Outbreak 8)	8	5	5	1	Contact, ingestion, inhalation	Nausea (5), vomiting (4), diarrhea (4), abdominal cramps (2), anorexia (1)	Fever (4), headache (4), dizziness (1), fatigue (3), malaise (1), back pain (1)	Skin irritation (6), rash (3)	Earache (2)	Confusion (3), neurologic symptoms (3)	Respiratory symptoms (5), cough (2), wheezing (1), chest tightness (1)
Ohio (Outbreak 9)	2	0	0	0	Contact, ingestion	Diarrhea (2), vomiting (2)					
Washington (Outbreak 10)	3	2	0	0	Unknown	Gastroenteritis (3)	Fever (1)				
Washington (Outbreak 11)	4	1	0	1	Ingestion	Gastroenteritis (3)		Dermatologic symptoms (1)	Ear symptoms (1)		Respiratory symptoms (1)

* Multiple levels of health care might have been accessed by a person (e.g., used emergency department and was hospitalized). No deaths were reported.

† Route(s) of exposure reported for the outbreak via the National Outbreak Reporting System.

§ Multiple health effects might be reported by a person. Information about each symptom might not have been available for all persons in an outbreak.

¶ Health-care information is unknown for two persons in this outbreak.

** Dog and fish illness or death also reported.

†† Health-care information is unknown for one person in this outbreak.

§§ Dog, fish, and bird illness or death also reported.

**TABLE 3:** Water quality indicators, by harmful algal bloom–associated waterborne disease outbreak — United States, 2009– 2010

Outbreak (by state)	Water quality indicators*					

	Cyanobacteria	*Escherichia coli*	Anatoxin-a (*μ*g/L)	Cylindrospermopsin (*μ*g/L)	Microcystin (*μ*g/L)	Saxitoxin (*μ*g/L)
New York (Outbreak 1)	X	—	—	—	112.5	—
New York (Outbreak 2)	—	—	—	—	—	—
New York (Outbreak 3)	X†	>126 CFU/mL§	—	—	—	—
Ohio (Outbreak 4)	—	—	0.05–0.1	ND	4.6	ND
Ohio (Outbreak 5)	—	—	—	—	>1,000.0	—
Ohio¶ (Outbreak 6)	X**	—	ND	ND	0.2	0.03
Ohio¶ (Outbreak 7)	—	—	—	ND	20.8	ND
Ohio¶ (Outbreak 8)	—	—	15.0	9.0	>2000.0	0.09
Ohio (Outbreak 9)	—	—	0.2	0.3	0.3	ND
Washington (Outbreak 10)	—	—	—	—	<6.0††	—
Washington (Outbreak 11)	—	—	—	—	—	—

**Abbreviations:** ND = not detected (Water test results indicated that the toxin was not present in water samples or that the concentration was below the level of detection); CFU = colony forming unit.

* Water quality indicators reported to the National Outbreak Reporting System or the Harmful Algal Bloom-Related Illness Surveillance System: identification of one or more cyanobacterial genera in lake water sample, *Escherichia coli,* and maximum cyanotoxin concentrations (i.e., within 1 day of outbreak exposure period).

† Both exposures occurred on a single day. *Microcystis* was identified by microscopy 3 days after the date of the exposure.

§ *E. coli* measurements exceeded 126 CFU/100 mL before, during, and after the exposure period. Reported levels were 328 CFU/100 mL (4 days prior), 488 CFU/100 mL (2 days prior), 152 CFU/100 mL (day of exposure), 248 CFU/100 mL (1 day after), and 222 CFU/100 mL (3 days after).

¶ Exposure occurred at a lake that was a water source for a public water system. Cyanotoxin analysis of finished water samples indicated that algal toxins were not present or that concentrations were below the limit of detection.

** Mixed toxigenic cyanobacteria bloom that included abundant cyanobacteria in succession: initially *Anabaena* spp., then *Cylindrospermopsis raciborskii*, *Aphanizomenon* spp., and *Planktolyngbya limnetica*. Four days after the date of last exposure, microcystin was not detected, saxitoxin was measured at 0.05 *μ*g/L, and anatoxin-a was measured at 0.05–0.1 *μ*g/L.

†† Microcystin measurements were >6 *μ*g/L during the week before the outbreak.

## Footnotes

* The following states received grants to participate in HABISS: Florida, Iowa, Maryland, Massachusetts, New York, Oregon, South Carolina, Virginia, Washington, and Wisconsin.

† Two criteria must be met for a health event to be defined as an outbreak associated with recreational water: two or more persons must be linked epidemiologically, and the epidemiologic evidence must implicate recreational water as the probable source of illness. The reporting form and guidance for reporting via NORS are available at http://www.cdc.gov/nors. Outbreaks can be voluntarily reported to CDC by public health agencies in U.S. states, the District of Columbia, Guam, Puerto Rico, the Republic of the Marshall Islands, the Federated States of Micronesia, the Commonwealth of the Northern Mariana Islands, the Republic of Palau, and the U.S. Virgin Islands.

§ Based on information for 10 outbreaks; data missing for one outbreak.

¶ More than one type of health effect or exposure could be reported by a single person.

** Additional information available at http://water.epa.gov/scitech/swguidance/standards/criteria/health/recreation/upload/factsheet2012.pdf.

†† One outbreak in New Hampshire (August 2001) in which *Oscillatoria* was suspected and isolated from a lake in a state park, and gastrointestinal symptoms were reported in 42 children; and two outbreaks in Nebraska (July 2004) involving microcystin from a lake, in which respiratory and dermatologic symptoms were reported in 22 persons. Additional information available at http://www.cdc.gov/healthywater/surveillance/rec-water-surveillance-reports.html.

§§ Data available at http://www.census.gov/compendia/statab/2012/tables/12s1249.pdf.
